# A randomized phase III double-blind placebo-controlled trial of first-line chemotherapy and trastuzumab with or without bevacizumab for patients with HER2/neu-positive metastatic breast cancer: a trial of the ECOG-ACRIN Cancer Research Group (E1105)

**DOI:** 10.1007/s10549-024-07417-4

**Published:** 2024-07-05

**Authors:** Jessica Mezzanotte-Sharpe, Anne ONeill, Ingrid A. Mayer, Carlos L. Arteaga, Ximing J. Yang, Lynne I. Wagner, David Cella, Neal J. Meropol, R. Katherine Alpaugh, Thomas J. Saphner, Robert E. Swaney, Karen L. Hoelzer, William J. Gradishar, Vandana G. Abramson, P. Kothai Sundaram, Shamim Z. Jilani, Edith A. Perez, Nancy U. Lin, Mohammad Jahanzeb, Antonio C. Wolff, George W. Sledge, Sonya A. Reid

**Affiliations:** 1 Vanderbilt University Medical Center, 2220 Pierce Avenue, 777 PRB, Nashville, TN 37232-6307 USA; 2 Dana-Farber Cancer Institute, Boston, MA USA; 3 UT Southwestern/Simmons Cancer Center, Dallas, TX USA; 4 Northwestern University, Chicago, IL USA; 5 Wake Forest University Health Sciences, Winston Salem, NC USA; 6 Case Western Reserve University, Cleveland, OH USA; 7 Fox Chase Cancer Center, Philadelphia, PA USA; 8 Aurora Health Care- Aurora Cancer Care, Two Rivers, Wl USA; 9 Denver Health Medical Center, Denver, CO USA; 10 Memorial Medical Center, Springfield, IL USA; 11 Columbus NCORP, Columbus, OH USA; 12 Good Samaritan Hospital, Dayton, OH USA; 13 Mayo Clinic, Jacksonville, FL USA; 14 University of Miami, Deerfield Beach, FL USA; 15 Johns Hopkins University, Baltimore, MD USA; 16 Stanford Cancer Institute, Stanford, CA USA

**Keywords:** Breast cancer, Trastuzumab, Chemotherapy, Bevacizumab

## Abstract

**Background:**

In 2008, bevacizumab received accelerated Food and Drug Administration (FDA) approval for use in human epidermal growth factor receptor 2 (HER2)-negative metastatic breast cancer (MBC). Based on the pre-clinical and preliminary clinical activity of the trastuzumab and bevacizumab combination, ECOG-ACRIN E1105 trial was developed to determine if the addition of bevacizumab to a chemotherapy and trastuzumab combination for first-line therapy would improve progression-free survival (PFS) in patients with HER2-positive MBC.

**Findings:**

96 patients were randomized to receive standard first-line chemotherapy and trastuzumab with or without bevacizumab between November 2007 and October 2009, and 93 began protocol therapy. Induction therapy was given for 24 weeks, followed by maintenance trastuzumab with or without bevacizumab. 60% (56/93) began carboplatin and 74% (69/93) completed 6 cycles of induction therapy. Primary endpoint was PFS. Median PFS was 11.1 and 13.8 months for placebo and bevacizumab arms, respectively (hazard ratio [HR] 95%, Confidence Interval [Cl] for bevacizumab vs. placebo: 0.73 [0.43–1.23], p = 0.24), and at a median follow-up of 70.7 months, median survival was 49.1 and 63 months (HR [95% Cl] for OS: 1.09 [0.61–1.97], p = 0.75). The most common toxicities across both arms were neutropenia and hypertension, with left ventricular systolic dysfunction, fatigue, and sensory neuropathy reported more frequently with bevacizumab.

**Conclusions:**

In this trial, the addition of bevacizumab did not improve outcomes in patients with metastatic HER2-positive breast cancer. Although the trial was underpowered due to smaller than anticipated sample size, these findings corroborated other clinical trials during this time.

Clinical Trial Information: NCT00520975

## Introduction

In 2008, bevacizumab received accelerated Food and Drug Administration (FDA) approval for use in human epidermal growth factor receptor 2 (HER2)-negative metastatic breast cancer (MBC). This was based off the E2100 study, which showed a combination of paclitaxel plus bevacizumab significantly prolonged progression-free survival (PFS) in treatment-naïve patients with MBC [1]. By 2011, however, the FDA withdrew its approval for bevacizumab due to the lack of evidence of OS benefit and concern for unacceptable toxicity [2].

During this time period, the interest in the use of bevacizumab in HER2-positive breast cancer was also high due to studies demonstrating association between HER2 amplification and increased vascular endothelial growth factor (VEGF) in breast cancer [3–5]. Additionally, a phase II trial at the time showed combining bevacizumab with trastuzumab in the treatment of HER2-positive MBC was both clinically feasible and active in the absence of chemotherapy [6]. Based on the pre-clinical and preliminary clinical activity of the trastuzumab and bevacizumab combination, E1105 was developed to determine if the addition of bevacizumab to first-line chemotherapy and trastuzumab would improve PFS in patients with HER2-positive MBC.

## Materials and methods

### Participants

Patients ≥ 18 years with histologically confirmed HER2-positive MBC, ECOG performance status of 0 or 1, adequate hematological, neurological, cardiac and end-organ function, and no prior systemic therapies were considered for enrollment [7]. Prior taxane and trastuzumab were allowed if given > 12 months prior to recurrence. The study was coordinated by the ECOG-ACRIN Cancer Research Group (ECOG-ACRIN), in collaboration with Radiation Therapy Oncology Group (RTOG), North Central Cancer Treatment Group (NCCTG), Cancer and Leukemia Group B (CALGB), Southwest Oncology Group (SWOG), National Surgical Adjuvant Breast and Bowel Project (NSABP), and Cancer Trials Support Unit (CTSU). Written informed consent was obtained from all patients before enrollment.

### Treatment

**Fig. 1:**
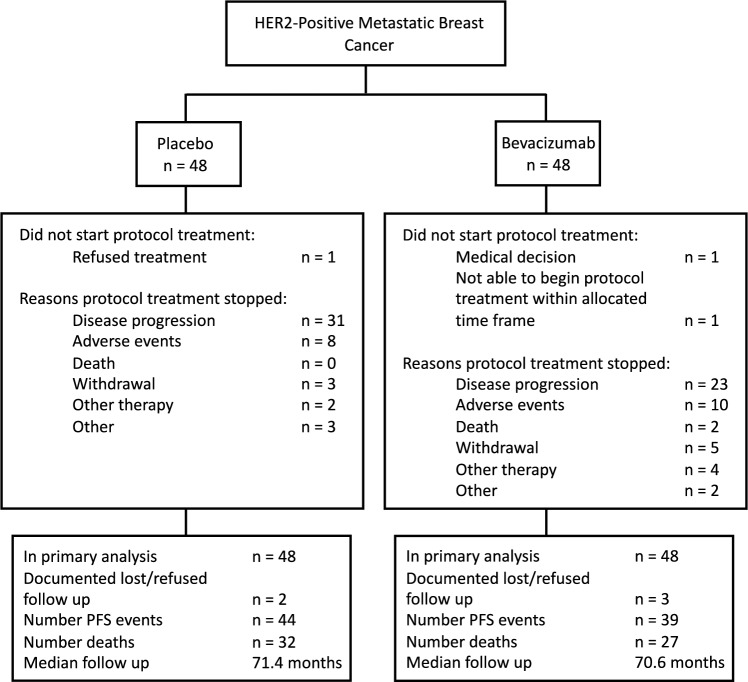
Study consort diagram

Patients were randomized to receive standard first-line induction chemotherapy (paclitaxel 90 mg/m2 IV weekly × 3 every 4 weeks [6 cycles] or paclitaxel 80 mg/m2 IV weekly × 3 every 4 weeks + carboplatin AUC 2 IV weekly × 3 every 4 weeks [6 cycles]) with trastuzumab (2 mg/kg IV weekly [after initial loading dose of 4 mg/kg] for 6 cycles) and placebo (PLAC) or bevacizumab (BEV) (10 mg/kg IV every 2 weeks for 24 weeks [6 cycles]), followed by maintenance trastuzumab (6 mg/kg IV every 3 weeks) and BEV or PLAC (15 mg/kg IV every 3 weeks) until disease progression, severe adverse event, pregnancy, withdrawal, or death. Concurrent endocrine therapy was not allowed during study treatment. Participants were allowed to discontinue chemotherapy and proceed to maintenance therapy. If trastuzumab or bevacizumab was discontinued, chemotherapy could continue. A schematic of the trial is provided in Fig. 1.

### Assessments

Tumor (Computer tomography/ bone scan) and cardiac (echocardiogram or MUGA) assessments were performed at baseline, every 3 months, and 3 months post-treatment. Tumor assessments continued until first progression. Complete blood counts were assessed prior to every cycle. Quality of life (QOL) assessments (FACIT-Fatigue Subscale, FACT/NCCN Breast Symptom Index, FACT/GOG-Ntx, FACT-G item GP5) were completed at baseline, end of cycles 3 and 6 induction, cycle 5 maintenance, 12 months post-randomization, and annually to 60 months post-randomization.

### Statistical considerations and endpoints

Progression-free survival (PFS) was the primary endpoint and was defined as time from randomization to first disease progression via RECIST 1.0, new second breast primaries, or to death from any cause. Blinded treatment assignments were made in permuted blocks in a 1:1 fashion to PLAC or BEV. Randomization was stratified by prior adjuvant trastuzumab use (yes, no), prior taxane use in the adjuvant or neoadjuvant setting (yes, no), disease-free interval (≤ 24 months, > 24 months), and planned carboplatin (yes, no). The accrual goal was 416 patients where 301 PFS events provided 86% power to detect a 30% reduction in the failure hazard rate. The trial was monitored by the ECOG-ACRIN DSMC, including a prespecified cardiac stopping rule for high rates of clinical CHF in BEV.

Secondary endpoints included overall survival (OS), defined as time from randomization date to death from any cause. The Kaplan–Meier method was used to estimate time-to-event distributions. Cox proportional hazards models were used to estimate hazard ratios and test for significance. Toxicities were graded according to CTCAE version 3.0. Cardiac safety profiles included clinical congestive heart failure (symptomatic decline in LVEF to below the lower limit of normal or symptomatic diastolic dysfunction). Baseline characteristics are reported among 95 of 96 with baseline information available, specific treatment information is reported among 93 of 96 patients who began protocol therapy, and best response, PFS, and OS are analyzed on an intent-to-treat basis.

## Results

### Baseline characteristics

Between November 9, 2007, and October 28, 2009, 96 patients with HER2-positive MBC were enrolled. Due to slow accrual toward the accrual goal of 416 patients, the trial was closed after October 2009. Table 1 provides a summary of baseline characteristics.

**Table 1:** Patient demographics and disease characteristics (n = 95*; n(%) shown unless otherwise specified)

	A (Placebo; PLAC)	B (Bevacizumab; BEV)	Total
	n = 48	n = 48	n = 96
*Race*			
White	41 (85)	37 (77)	78 (81)
Black	6 (13)	7 (15)	13 (14)
Asian	1 (2)	2 (4)	3 (3)
Native Hawaiian	0	1 (2)	1 (1)
Missing	0	1	1
*Age*			
Median (min, max)	55 (27, 77)	55 (33,76)	55 (27,77)
*Menopausal Status*			
Postmenopausal	33(69)	34 (71)	67 (70)
Premenopausal	13 (27)	13 (27)	26 (27)
Perimenopausal	1 (2)	1 (2)	2 (2)
Missing	1		1
*ECOG PS*			
0	33 (69)	31 (65)	64 (67)
1	14 (29)	17 (35)	31 (32)
Missing	1		1
*ER Status*			
Negative	18 (38)	20 (42)	38 (40)
Positive	29 (60)	28 (58)	57 (59)
Missing	1		1
*Site of Involvement*			
Local–regional	32 (67)	31 (65)	63 (66)
Ipsilateral supraclavicular	7 (15)	6 (13)	13 (14)
Opposite breast	1 (2)	2 (4)	3 (3)
Distant lymph nodes	13 (27)	21 (44)	34 (36)
Bone	26 (54)	16 (33)	42 (44)
Bone Marrow	2 (4)	–	2 (2)
Lung	17 (35)	17 (35)	34 (16)
Liver	27 (56)	22 (46)	49 (51)
Pleura	2 (4)	2 (4)	4 (4)
Other	1 (2)	2 (4)	3 (3)
*Prior Hormonal Therapy*			
No	34 (71)	33 (69)	67 (70)
Yes	13 (27)	15 (31)	28 (29)
In adjuvant setting	11	11	22
In metastatic setting		4	4
Both	2		2
Missing	1		1
*Prior RT*			
No	33 (69)	31 (66)	64 (67)
Yes	14 (29)	16 (34)	30 (31)
Missing	1		1
*Prior Taxane in Adjuvant or Neo-adjuvant Setting*			
No	38 (79)	35 (73)	73 (76)
Yes	9 (19)	13 (27)	22 (23)
Missing	1		1
*Prior Doxorubicin in Adjuvant or Neo-adjuvant Setting*			
No	30 (63)	35 (73)	65 (68)
Yes	17 (35)	13 (27)	30 (31)
Missing	1		1
*Prior Trastuzumab in Adjuvant Setting*			
No	41 (85)	40(83)	81 (84)
Yes	6 (13)	8 (17)	14 (15)
Missing	1		1
*Prior Chemotherapy (other) in Adjuvant Setting*			
No	35 (73)	39 (81)	74 (77)
Yes	12 (25)	9 (19)	21 (22)
Missing	1		1

*Missing On-Study Form for 1 patient

Among the patients who began treatment in the PLAC (n = 47) and BEV (n = 46) arms, 64% (30/47) and 57% (26/46) began the optional carboplatin, and 72% (34/47) and 76% (35/46) completed 6 cycles of induction therapy, respectively. The median number of cycles for maintenance therapy (n = 63) was 8 and 16 for the PLAC and BEV arms. Disease progression (66% [31/47] and 50% [23/46]) and adverse events (15% [7/47] PLAC and 22% [10/46] BEV) were the most common reasons for discontinuing treatment.

### Clinical efficacy and secondary endpoints

**Fig. 2:**
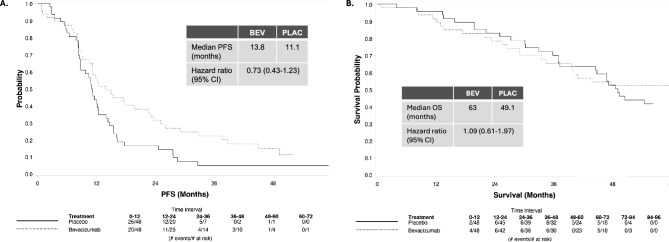
PFS and OS. **A** Median PFS was 11.1 months in the PLAC (placebo) arm and 13.8 months in the BEV (bevacizumab) arm, p = 0.24. **B** Median OS was 49.1 months in the PLAC arm and 63 months in the BEV arm (figure truncated at 60 months), p = 0.75. CI = confidence interval

The best overall response rate (CR + PR) was 54% (26/48) and 61% (29/48) in PLAC and BEV, respectively (Table 2). There was no statistically significant difference in PFS: median PFS was 11.1 and 13.8 months; hazard ratio (HR) (95% Confidence Interval [Cl]) for BEV vs. PLAC: 0.73 (0.43–1.23), p = 0.24 (Fig. 2A). With n = 96 patients and 83 PFS events, the power to detect the original target difference between arms was only 37%. At a median follow-up of 70.7 months, median overall survival was 49.1 and 63 months; (HR [95% Cl] 1.09 [0.61–1.97], p = 0.75) (Fig. 2B (truncated at 60 months)).

**Table 2:** Clinical response and outcomes

	A (Placebo; PLAC)	B (Bevacizumab; BEV)	Total
	n = 48	n = 48	n = 96
*Best Overall Response*	n (%)	n (%)	n (%)
CR	4(8)	6(13)	10(10)
PR	22(46)	23(48)	45(47)
Stable	13(27)	9(19)	22(23)
PD	6(13)	5(10)	8(8)
Not evaluable	3(6)	5(10)	8(8)
*Progression-Free Survival*			
# of events	44	39	83
Median in months(95% CI)	11.1(8.7–12.4)	13.8(10.6–22.5)	
HR* (95%CI), p value**			
Univariate		0.73(0.43–1.23),0.24	
Multivariable***		1.09(0.61–1.97),0.75	
*Overall Survival*			
# of events	32	27	59
Median Follow-up (months)	71.4	70.6	70.7
Median in months(95% CI)	49.1(37.2–67.4)	62.9(34.4–72.8 +)	
HR* (95%CI), p value**			
Univariate		1.09(0.61–1.97),p = 0.75	
Multivariable***		1.12(0.62–2.04),p = 0.69	

*Bevacizumab vs. placebo

**P-values are two-sided and are based on stratified test using stratification factors at randomization

***Adjusted for Age, ERPR, Primary surgery

+ last censored observation as upper limit not reached

### Overall toxicity and cardiac safety

Toxicity incidence, defined as ≥ grade 3 and experienced by ≥ 2 patients, occurred in 47% (22/47) of PLAC and 67% (30/45) of BEV patients (Table 3). The most frequent ≥ grade 3 toxicities across both arms were neutropenia (6.4%, 6.7%) and hypertension (10.6%, 13.3%). Left ventricular systolic dysfunction (0%, 8.9%), fatigue (2.1%, 11.1%), and sensory neuropathy (6.4%, 11.1%) occurred more frequently in the BEV arm. At Cycle 6 Induction, more BEV patients reported fatigue compared to PLAC (FACIT Fatigue Scale: mean scores: 30.2 and 37.0, p = 0.02; FACT-G item GP5 [“I am bothered by sided effects of treatment”]: mean scores: 2.5 and 3.2, p < 0.01). One patient treated with bevacizumab died from treatment-related catheter infection (Table 3). Clinical congestive heart failure occurred in 1 PLAC and 4 BEV patients.

**Table 3:** Toxicity incidence (includes ≥ grade 3 and experienced in ≥ 2 patients)*

Toxicity Type	Treatment arm							
	A (Placebo, n = 47)				B (Bevacizumab, n = 45)			
	Grade	3	4	5	Grade	3	4	5
		(n, %)	(n, %)	(n, %)		(n, %)	(n, %)	(n, %)
Allergic reaction		1 (2)	–	–		4 (9)	–	–
Neutrophils		–	3 (6)	–		–	3 (7)	–
Hypertension		5 (11)	–	–		6 (13)	–	–
Left ventricular systolic dysfunction		–	–	–		3 (7)	1 (2)	–
Fatigue		1 (2)	–	–		4 (9)	1 (2)	–
Wound—non-infectious		–	–	–		2 (4)	–	–
Anorexia		1 (2)	–	–		2 (4)	–	–
Dehydration		1 (2)	1 (2)	–		2 (4)	–	–
Diarrhea w/o prior colostomy		4 (9)	–	–		3 (7)	–	–
Nausea		2 (4)	–	–		2 (4)	–	–
Vomiting		2 (4)	–	–		3 (7)	–	–
Infection Gr0-2 neut, catheter		–	–	–		–	–	1 (2)^
InfectionGr0-2 neut, skin		1 (2)	–	–		1 (2)	–	–
Hyperglycemia		1 (2)	–	–		1 (2)	1 (2)	–
Proteinuria		1 (2)	–	–		4 (9)	–	–
Nonneuropathic generalized weakness		1 (2)	–	–		2 (4)	–	–
Neuropathy-motor		2 (4)	–	–		3 (7)	–	–
Neuropathy-sensory		3 (6)	–	–		5 (11)	–	–
Cough		1 (2)	–	–		1 (2)	–	–
Renal failure		2 (4)	–	–		–	–	–
Thrombosis/thrombus/embolism		–	1 (2)	–		1 (2)	1 (2)	–

* Only patients who began protocol treatment are included in this summary, and incidence rates are reported based on the number of patients in each treatment arm (One patient on Arm A did not begin protocol treatment, and two patients on Arm B did not begin protocol treatment. In addition, one patient on Arm B had no toxicities reported.) Worst event reported per patient based on CTCAE version 3.0 with the following attributions: possibly, probably, or definitely related to treatment. AE reporting in the trial was limited to ≥ grade 4 hematologic events, ≥ grade 2 non-hematologic events for a select group of AEs, and ≥ grade 3 non-hematologic events otherwise

^patient died within 30 days of ending protocol treatment

## Discussion

During the enrollment of the presented study, several factors resulted in low patient accrual and early study closure in 2009, including new treatment paradigms with decreased metastatic recurrence following neoadjuvant treatment with trastuzumab [8]. Additionally, there were new drug approvals in the metastatic setting such as lapatinib [9] and excitement regarding other HER2-targeted agents in the metastatic setting (such as pertuzumab and trastuzumab-emtansine). Due to low accrual, the study was underpowered, and no significant difference in clinical outcomes was observed between treatment arms. The safety profiles for bevacizumab and trastuzumab were consistent with prior phase I, II, and III trials. Cardiac toxicity is a known side effect of trastuzumab; however, previous studies found the toxicity was reversible, unlike doxorubicin-induced cardiomyopathy [10, 11]. We saw few overall cardiac adverse events from bevacizumab in addition to the hallmark hypertension associated with anti-angiogenic drugs [12].The results from this trial corroborated the 2011 FDA decision to remove bevacizumab as a recommended therapeutic option for patients with breast cancer.

Since the closure of E1105, other clinical trials have explored the combination of HER2-targeted and anti-angiogenic therapies. A phase II single-arm trial of bevacizumab, trastuzumab, and capecitabine showed clinical activity as first-line therapy for patients with HER2-positive MBC, with no unexpected toxicities, and a median time to progression of 14.5 months (95% Cl 10.5 months to NR) [13]. The AVEREL study randomized 424 first-line patients to trastuzumab/docetaxel with or without concomitant bevacizumab [14]. Despite a trend favoring bevacizumab PFS (median 13.7 vs. 16.5 months; HR 0.82, log-rank P = 0.078), no difference was observed in overall survival [14]. The BETH study randomized 3509 patients with HER2-positive early-stage breast cancer to receive standard chemotherapy/trastuzumab with or without bevacizumab for 1 year of adjuvant therapy [15]. After 38 months of follow-up, there was no statistically significant difference between treatment arms (92% IDFS rates in both groups). Currently, the majority of recent studies using bevacizumab to treat breast cancer are in the HER2-negative patient population or are in subsets of HER2-positive and -negative patients with specific types of refractory disease [16, 17].

More recently, the major focus on treating refractory HER2-positive MBC lies in developing new HER2-targeted antibody–drug conjugates, combinations of CDK4/6 or PI3K/Akt inhibitors with these agents as well as with endocrine therapies, and combinations of different immunotherapy agents with HER2-targeted therapies (18). Despite the strong pre-clinical rationale for combining HER2-targeted therapies with anti-angiogenic drugs, there was no overall benefit, and there was added toxicity of combining bevacizumab with trastuzumab and chemotherapy, aligning with the 2011 FDA decision to remove the recommendation for the use of bevacizumab in all breast cancers. Despite advances in the adjuvant and metastatic setting made over the past decades, approximately, 15–20% of patients with early HER2-positive breast cancer still relapse after curative therapy, and HER2-positive MBC remains incurable. Therefore, new therapeutic approaches are still necessary for this disease.

## Data Availability

All study data may be requested from the corresponding author and/or ECOG-ACRIN research group upon reasonable request.
